# A systematic review of dalbavancin efficacy as a sequential therapy for infective endocarditis

**DOI:** 10.1007/s15010-024-02393-9

**Published:** 2024-09-26

**Authors:** Gabriele Maria Leanza, Emanuele Rando, Federico Frondizi, Eleonora Taddei, Francesca Giovannenze, Juan P. Horcajada, Giancarlo Scoppettuolo, Carlo Torti

**Affiliations:** 1 Dipartimento di Sicurezza e Bioetica - Sezione di Malattie Infettive, Università Cattolica del Sacro Cuore, Rome, Italy; 2 Dipartimento di Scienze Mediche e Chirurgiche, Fondazione Policlinico Universitario Agostino Gemelli IRCCS, Rome, Italy; 3 Hospital del Mar. Hospital del Mar Research Institute, Pompeu Fabra University (UPF), Barcelona, SpainCIBER of Infectious Diseases (CIBERINFEC CB21/13/00002), Institute of Health Carlos III, Madrid, Spain

**Keywords:** Dalbavancin, Long acting antibiotics, Infective endocarditis, Sequential therapy, Gram-positive

## Abstract

**Introduction:**

Dalbavancin is an antibiotic characterized by an extended half-life and efficacy against methicillin-resistant *Staphylococci*. Currently, there are only narrative reviews summarizing the evidence about the use of dalbavancin for infective endocarditis (IE), many of which are focused primarily on its use as consolidation therapy. For this reason, we conducted a systematic review to describe the clinical efficacy and the safety of dalbavancin in IE treatment.

**Methods:**

We searched for available evidence using the MEDLINE (PubMed), Embase, Scopus, Cochrane Library and Web of Science libraries, with no restrictions regarding the publication year. The risk of bias was performed using the Cochrane ROBINS-I tool for the comparative studies and the Newcastle-Ottawa Scale for descriptive studies.

**Results:**

Nine studies were included. All of them were observational. Native valve endocarditis was the most common kind of IE found in the studies’ populations (128/263, 48.7%), followed by prosthetic valve endocarditis, and cardiovascular implantable electronic device-related endocarditis. Coagulase-negative *Staphylococci* were the most common pathogens isolated (83/269, 30.1%), followed by *S. aureus*, *Enterococci* spp and *Streptococci* spp. Five out of nine studies documented a clinical failure rate of less than 10%. Dalbavancin showed a favourable safety profile. Dalbavancin appears to be a promising option for the consolidation therapy of IE. However, further studies comparing dalbavancin with standard of care are needed.

**PROSPERO registration number:**

CRD42023430032.

**Supplementary Information:**

The online version contains supplementary material available at 10.1007/s15010-024-02393-9.

## Introduction

IE remains a significant clinical challenge due to its complex management and high morbidity and mortality [1, 2]. In this context, dalbavancin, a novel antibiotic belonging to the lipoglycopeptide class, has recently received attention for treating Gram-positive bacterial infections, gradually finding broader applications in clinical practice. These include the treatment of conditions such as IE, vascular graft infections (VGIs), bone and joint infections (BJIs), and prosthetic joint infections (PJIs) [3].

Preclinical and in vitro studies have previously highlighted dalbavancin’s potential as an effective therapeutic option for endocarditis caused by both methicillin-susceptible *S. aureus* (MSSA) and methicillin-resistant *S. aureus* (MRSA), as well as other Gram-positive bacteria, including CoNS and streptococcal species [4–7]. Dalbavancin is an antibiotic with an extended half-life ranging from 333 to 405 h [8].

Dalbavancin could be a valuable option as sequential therapy for the infections requiring a prolonged antibiotic treatment. Its use has the potential to substantially decrease hospital length of stay and associated costs for patients afflicted with the aforementioned infections [9].

Given the increasing clinical use and the urge for more robust scientific evidence to support this drug utilization, numerous studies have been undertaken. While some of them are still ongoing, others have already yielded valuable insights, focusing on improving the definitions of pharmacokinetics to enhance the use of this drug in outpatient parenteral antibiotic therapy (OPAT) for IE [10, 11].

In this regard, the dalbavancin off-label applications, including those related to IE, have recently been discussed in narrative reviews [12, 13]. Yet, a more comprehensive and focused literature review on dalbavancin as consolidation therapy for endocarditis is still lacking. A systematic approach to reviewing the current evidence is needed to bring knowledge together and guide clinical practice and future research. For this reason, we conducted a systematic review on dalbavancin use for treating IE. Our study aims to systematically retrieve current evidence regarding clinical efficacy and safety of dalbavancin in the treatment of IE.

## Methods

We registered this systematic review protocol in the PROSPERO database (protocol number CRD42023430032, published on 09 June 2023). We followed the Preferred Reporting Items for Systematic Reviews and Meta-analysis (PRISMA) checklist for this review (Supplementary materials). We performed the article screening and full-text review through Rayyan, a web and mobile app for systematic reviews [14]. We used pre-defined Excel spreadsheets for data extraction and bias assessment of the included studies.

### Systematic search and libraries

We searched for available evidence using the MEDLINE (PubMed), Embase, Scopus, Cochrane library and Web of Science libraries. We performed the final search on 19 May 2023. We composed the search string using the appropriate words referring to dalbavancin and IE. We reported the complete strings in Supplementary materials. We did not use any filters, limits, or language restrictions. Authors have followed the MEDLINE library monthly until 01/02/2024 to see if additional articles regarding dalbavancin and IE were published.

### Eligibility study criteria and work process

To consider suitable studies, we used the following inclusion criteria: (i) randomized controlled trials, non-randomized intervention studies, observational studies including case series involving a minimum of 15 patients with IE; (ii) adult patients ≥ 18 years old; (iii) studies dealing with dalbavancin; (iv) studies published in peer-reviewed journals. We did not impose language and date restrictions.

We followed these exclusion criteria: (i) case reports, studies including a population with less than 5 patients with IE treated with dalbavancin; preclinical studies, animal and in vitro studies, narrative reviews, systematic reviews and meta-analysis; (ii) paediatric or adolescent populations < 18 years old, pregnant or breastfeeding women; (iii) studies not dealing with dalbavancin for the treatment of endocarditis; (iv) unpublished studies, preprints, study protocols, conference papers, and abstracts, studies published without a peer-reviewed process.

We manually removed all duplicates in the systematic review software. After this, two independent and blind reviewers (G. M. L. and F. F.) screened and full text reviewed all articles. A third author (E. R.) resolved conflicts between them when present in both phases. Once included studies were retrieved, two reviewers (G. M. L. and F. F.) performed the data extraction. Disagreements were resolved by collegial discussion among three authors to reach consensus (G. M. L., F. F., and E. R.).

We extracted data regarding study identification characteristics, study design, population characteristics and number, involved pathogens and their microbiological profile, intervention characteristics and use, and outcome data when present, including 6-month mortality, 12-month mortality, recurrence of infection, adverse events, unplanned cardiac surgery, clinically evident embolic events, and clinical efficacy according to individual study definition.

### Bias assessment and synthesis of findings

Two authors (G. M. L. and F. F.) performed the quality assessment independently. Another author (E. R.) resolved disagreements between the first two authors through collegial discussion. Once consensus was reached, we assigned the bias judgement.

We used two quality assessment tools, since we only retrieved observational studies. For comparative studies, we selected the Cochrane ROBINS-I tool to comprehensively evaluate all bias domains given the non-randomized nature of these papers. In contrast, we used the Newcastle-Ottawa Scale for descriptive studies to discriminate whether the study population was representative and to address unsolvable questions of the ROBINS-I tool, considering the lack of a comparative intervention.

Given the presence of descriptive and cohort studies, we judged a meta-analysis unfeasible and non-informative. For this reason, we provided a narrative synthesis of the findings from the included studies. We included textual descriptions and tables summarizing the study characteristics and results, discussing the overall level of evidence certainty.

## Results

### Included studies

**Fig. 1:**
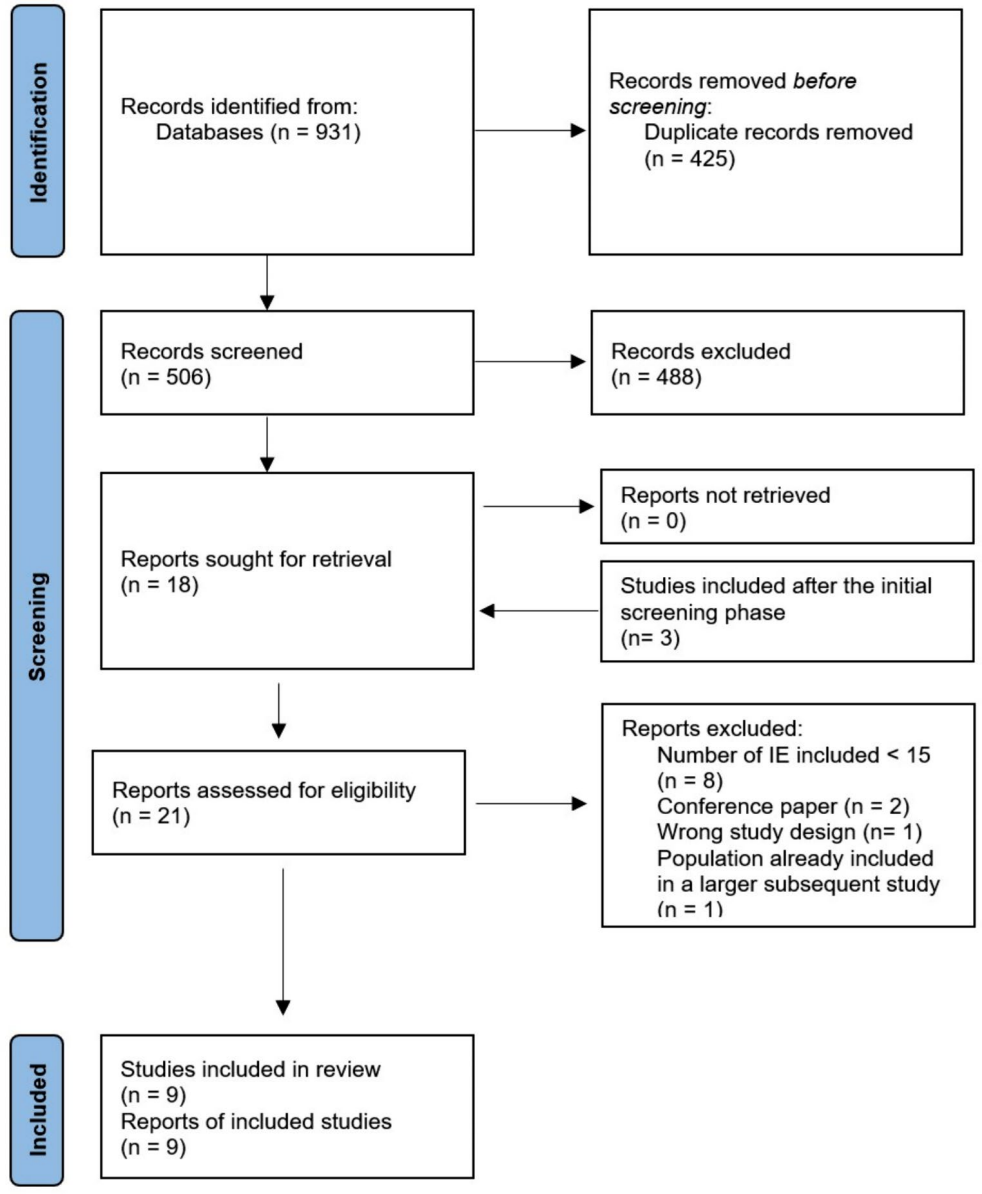
PRISMA flow-chart

The systematic review flow diagram is shown in Fig. 1. Our search found 931 studies. After duplicates removal, 506 studies underwent the screening and full-text steps. Among these, 18 studies were initially assessed for eligibility. Additionally, three more studies published after the initial library searches were identified and deemed relevant to the research question, meeting the eligibility criteria [15–17]. Of the 21 reports that underwent the full-text review, as shown in the PRISMA flow-chart and in Table S4 of the supplementary materials, eight were excluded due to an insufficient number of patients affected by IE, two due to wrong publication type (conference papers), one due to the wrong study design and one because its population was lately included in a larger subsequent study.

Finally, nine studies were included. All of them were observational, with the majority (8/9) characterized by a retrospective data collection. Only three out of nine featured a comparator group to evaluate dalbavancin’s effectiveness. The majority of the included studies originated from Spain (4), two studies were performed in France, two studies in Austria, and 1 in the USA. The sample sizes varied across all studies. The study with the largest cohort gathered 124 patients treated with dalbavancin as consolidation therapy, the smallest one was composed by only 8 patients with IE treated with dalbavancin.

### Bias assessment

The results of the bias assessment phase are shown in Table 1. Among the six non-comparative observational studies, four achieved a rating of 6 out of 9 stars, and two studies achieved a rating of 5/9 stars, according to the New Castle-Ottawa Scale for cohort studies.

**Table 1:** Cohort studies included in the review

Author, year	Study period	Number of endocarditis	Endocarditis type	Pathogens	Time to first dalbavancin infusion (median)	Dalbavancin therapy duration/number of dalbavancin doses	Study outcome	Quality assesment
Tobudic, 2018 [21]	2015–2016	31 (4 lost at FU)	NVE 16, PVE 6, CIED 5	MSSA 9, CoNS 6, *Enterococcus* spp. 4, *Streptococcus* spp. 8	1 week	6 weeks of dalbavancin administration (median)	6-months clinical/microbiological failure: 7.4%	6/9
Dinh, 2019 [19]	2017–2018	19 (1 lost at FU)	NVE 8, PVE 10	CoNS 8, MSSA 3, MRSA 2, PId 3, E. faecalis 3, *S. gallloliticus* 1, *L. monocytogenes* 1	13 d	2 injections (median). One patient was under chronic suppressive therapy	Clinical failure at last visit (mean of 97.9 ± 99.7 d since first dose): 27.8%	5/9
Wunsch, 2019 [20]	2016–2019	25 (2 lost at FU)	NVE 15, PVE 6, CIED 4	NBCe 8, CoNS 5, PI 5, MSSA 2, MRSA 2, *Streptococcus* spp. 2, *Enterococcus* spp. 1	Not specified	2 doses (median)	90 d-clinical failure: 8.7% (2/23 patients with a complete FU)	6/9
Morata, 2022 [22]	2018–2019	38	Left-sided endocarditis 26 Right-sided endocarditis 12	MSSA 7, MRSA 3, CoNS 14, *Enterococcus* spp. 5	28 d	Mean: right heart IE 2.5 weeks, left heart IE 2 weeks 1 chronic therapy	Clinical failure: 2.6% (timing not specified)	6/9
Hidalgo-Tenorio, 2023 [14]	2016–2021	124 (1 lost at FU)	NVE 58, PVE 54, CIED 18	MSSA 28, MRSA 3, CoNS 48, *Enterococcus* spp. 27, *Streptococcus* spp. 18	Not specified	14 d of dalbavancin coverage (median)	12-months clinical failure: 4.8%	6/9
Courjon, 2023 [15]	2018–2020	21	PVE 17, NVE 4	Not specified for IE	Not specified for IE	Not specified for IE	30 d-clinical failure: 0% (for patients who completed the FU)	5/9

Regarding the three studies with a comparator group, one was evaluated as having a moderate risk of bias [18], while the other two were judged to be at serious risk of bias [17, 19] according to the ROBINS-I tool (Table 2). The studies conducted by de Pablo-Miró and Veve were judged at moderate risk of confounding bias due to their observational design. In contrast, the study conducted by Suárez was deemed to have a serious risk of confounding bias, primarily due to the absence of an appropriate analysis method that controlled for potential confounding domains. All three studies had minimal missing data. In the study conducted by Veve et *al*. on a population undergone OPAT for various infections, there were some concerns regarding selection bias. Notably, a higher percentage of patients in the dalbavancin group were discharged home (91% vs. 72%, *p* = 0.001), contrasting with the control group where patients were more likely to be transferred to an assisted care facility. This discrepancy might indicate a higher proportion of medically fit patients in the dalbavancin group. The authors note that this difference can be attributed to a larger percentage of patients without health insurance in the dalbavancin group (70% vs. 83%, *p* = 0.023). These aspects make it difficult to assess the bias direction. A detailed description of each bias domain is reported in the Supplementary Material.

**Table 2:** Studies with a control group included in the review

Author, year	Study period	*N* endocarditis	Endocarditis type	Pathogens	Time to first dalbavancin infusion (median)	Dalbavancin therapy duration/number of dalbavancin doses	Outcome	Risk of bias^a^
Veve, 2020 [18]	2016–2019	12	NVE 12	MRSA 9, MSSA 1, *E. faecalis* 1, NBC^c^ 1	11 d	6 weeks (median of expected dalbavancin concentration above MIC99)	90 d-overall-mortality: 16.7%	serious
De Pablo-Miró, 2021 [17]	2015–2019	8	NVE 7, CIED1	MSSA 3, *Streptococcus spp.* 3, CoNS 1, *Coynebacterium spp.* 1	11 d	5 doses (median)	30 d-Clinical failure: 50%	Moderate
Suárez, 2024 [16]	2015–2022	22	NVE 8, PVE 14	*S. aureus* 5, CoNS 2, *Enterococcus spp.* 12, *Streptococcus spp.* 3	Not specified	Not specified	12 months-Clinical failure: 22.7% (4 deaths and 1 recurrence)	Serious

### Populations of the included studies

#### Indications and isolated pathogens

Native valve endocarditis (NVE) was the most common IE found in the studies’ populations (128/263, 48.7%), followed by prosthetic valve endocarditis (PVE) (107/263, 40.7%), and cardiovascular implantable electronic device (CIED)-related endocarditis (28/263, 10.6%).

Microbiological causative agents of the IE cases were clearly reported in 8/9 studies. Considering the aggregated data, CoNS (83/269, 30.1%) and *S. aureus* (78/269, 29%) were the most common pathogens. Among the *S. aureus* group, we found 53 MSSA (67.9%) and 19 MRSA (24.4%). Other causative bacteria were *Enterococcus* spp. (53/269, 19.7%) and *Streptococcus* spp. (35/269, 13%). Only one study reported the dalbavancin MIC for the pathogens [20].

We identified two studies that included cases of IE with negative blood cultures [19, 21]. In particular, Wunsch et *al*. reported a cohort comprising eight cases of IE with negative blood cultures treated with dalbavancin; all of them had a positive 90-days clinical outcome defined in study as the absence of clinical, laboratory or microbiological evidence of persistent or recurring infection during the follow-up period [21].

#### Dosing and therapy regimens

We found only six patients in whom dalbavancin was administered from the beginning of IE therapy [20, 22, 23]. In contrast, in all remaining studies patients underwent dalbavancin therapy as a consolidation regimen subsequent to prior antibiotic treatments. Five out of nine clearly reported the number of patients who underwent surgical valve replacement. Among these five studies, we found 99/272 patients who received surgical treatment of IE.

Dalbavancin dosage varied considerably across the included studies. Looking at aggregate data, the most common dalbavancin regimen was a single 1500 mg dose (77 patients), followed by 1000 mg and 500 mg doses one week apart (29 patients), a single 1000 mg dose (25 patients), and a sequence of 1500 mg doses with a one-week interval between them (10 patients).

In only three studies, the precise number of patients receiving concomitant antibiotics was explicitly stated. Hidalgo-Tenorio et *al.* reported that, among their 124 subjects, 16 individuals (12.9%) were administered dalbavancin concurrently with other antibiotics, rifampicin being the most frequently prescribed medication in this context (10/16 patients, 62.5%), followed by levofloxacin (3/16, 18.8%), amoxicillin (2/16, 12.5%) and metronidazole (1/16, 6.3%) [15]. Among the 22 patients treated with dalbavancin as described by Suárez, two patients with PVE received rifampicin concurrently with dalbavancin [17]. In the cohort study conducted by Morata et *al.*, among the 38 cases of IE treated with dalbavancin, 7 patients were concomitantly prescribed antibiotics (18.4%). These regimens were not described [23]. In the study conducted by Courjon et *al*., the subgroup of vascular infections, including 21 cases of endocarditis and 1 case of arteritis, involved 14 patients who received dalbavancin in conjunction with another antibiotic. Consequently, a minimum of 13 out of 21 patients with IE underwent combination antibiotic therapy [16].

### Outcome

The outcomes of all studies are reported in Tables 1 and 2. Five out of nine studies documented a clinical failure rate of less than 10%. Most studies reported outcomes which were composite in nature, including assessments conducted by the same healthcare providers responsible for prescribing dalbavancin regarding the resolution of signs and symptoms. In the study conducted by Courjon et *al*., the authors reported a clinical success rate of 100%. Although it is worth noticing that the follow-up period was too short (30 days) for assessing IE outcomes [16]. Indeed, patients with a history of endocarditis sometimes experience recurrences and/or reinfections or may require additional cardiac surgeries even months after the initial diagnosis, which is why an adequate follow-up of at least six months is widely considered necessary [24].

Regarding mortality, some studies reported endocarditis-related mortality, while others reported all-cause mortality. Furthermore, there was considerable variation in the follow-up times across the studies. Consequently, presenting aggregated data on mortality or the clinical/microbiological success rate across the studies may not provide meaningful insights.

Among the eight patients with IE who were treated with dalbavancin in the study by De Pablo Miró, three individuals experienced clinical failure, and one patient died within 30 days from treatment initiation, although the cause of death was unrelated to IE. All of these four patients switched to dalbavancin very early, within less than 14 days of treatment with standard of care (SoC) [18].

Suárez et *al*. conducted the single comparative study focused on IE. Regarding clinical efficacy, no statistically significant differences were identified between the dalbavancin group and the SoC group (86% vs. 96%, *p* = 0.318). It is worth noting that the cohort size was substantially small (22 patients in the dalbavancin group vs. 47 in the SoC group), and the authors did not calculate the sample size needed to detect a statistically significant difference between the two groups. In the dalbavancin group 4 patients died and 1 patient went through a relapse within 12 months after the diagnosis of IE. Interestingly, among these five patients, four presented an IE caused by *E. faecalis* [17].

Tobudic et *al.* reported a case of microbiological failure in a patient with a CIED-related IE caused by MSSA with the development after 30 weeks of treatment with dalbavancin of an increased MIC of teicoplanin (16 mg/L) and vancomycin (2 mg/L). This failure occurred in the context of incomplete surgical source control. Moreover, in this retrospective cohort, dalbavancin was used as primary therapy in three outpatient parenteral antibiotic therapy (OPAT) cases: one NVE, one PVE, and one CIED-related IE. No relapses were observed during six months of follow-up, making this one of the few instances where dalbavancin was used as primary rather than consolidation therapy [22].

### Safety

In seven out of nine studies, the incidence rate of adverse events was below 10%. The most common adverse events reported in the included studies were asthenia, dizziness, pruritus, eosinophilia, nausea and vomiting [15, 16, 20, 22, 23]. Only 2 cases of *C. difficile* colitis were found among the populations described in the studies included in the review [15]. Courjon et *al.* described a cohort of 151 patients who underwent dalbavancin treatment for various indications. Among these patients, 67 individuals (44.4%) reported at least one adverse event. Within this group of 67 patients, 31 (20.5%) encountered a serious adverse event, and 7 (4.6%) experienced a fatal adverse event [16].

## Discussion

In our review, we identified nine studies, all observational in nature, comprising eight retrospective and one prospective observational study. They documented the utilization of dalbavancin in the context of IE. No clinical trial was found on the topic. Interestingly, only three out of the nine studies were specifically focused on IE [15, 17, 22], while the remaining encompassed patient cohorts treated with dalbavancin for various types of infections. This systematic review provides an overview of the clinical evidence for the use of dalbavancin in the management of IE.

A narrative review was previously published addressing the utilization of dalbavancin for the management of IE [13]. This review encompassed 16 studies. In line with the review by Fazili et *al*., most IE included in the studies involved a native valve. Concerning the etiological agents responsible for IE, Fazili et *al*. identified *S. aureus* as the most frequently documented microorganism in the studies they examined. In contrast, our review identified CoNS as the predominant species isolated.

This narrative review included all study types, comprising case reports and case series. In contrast, our systematic review excluded case reports. We only included case series featuring a larger sample of at least 15 patients. Furthermore, adhering to the guidelines outlined in the Cochrane Handbook for Systematic Reviews of Interventions [25], we sought additional data from the authors of studies where relevant information about the treatment of endocarditis with dalbavancin and their outcomes was missing. In this way we aimed for a comprehensive and rigorous review of the current evidence.

We found some limitations across the included studies. For instance, the dosage of dalbavancin exhibited significant variability across the studies included in the analysis. The variability in dosing regimens observed could be attributed to the lack of conclusive evidence concerning the appropriate dalbavancin dosage for the treatment of IE. Recently, an expert panel established the regimens for achieving adequate dalbavancin blood concentrations when a therapy duration longer than 2 weeks is required. In cases necessitating an extended treatment duration of up to 6 weeks, such as *S. aureus* NVE (4 weeks) or PVE (6 weeks), the expert panel recommends an initial 1500 mg dose, followed by a second 1500 mg dose administered after 8 to 15 days [11]. Nonetheless, within the studies encompassed in our review, the majority of dalbavancin treatments were initiated following prior intravenous antibiotic therapy (see Tables 1 and 2), and the median durations of these preceding treatments exhibited considerable variability across the diverse studies. This aspect contributes to the observed variability in dosing regimens within the included studies. It may be important to highlight that the included studies did not perform a subgroup analysis to evaluate outcomes based on the combination therapy regimen and the dalbavancin dosing schedule, which represents an additional limitation. In this regard, it could be interesting for future studies to assess variations in outcomes based on the therapeutic regimen and/or concomitant conditions, considering that these aspects may be influenced by the type of IE (e.g. PVE) and/or the presence of concomitant infection sites (e.g., spondylodiscitis).

Furthermore, the clinical outcomes of the included studies exhibited a lack of robustness. Notably, a significant proportion of the reported outcomes in these studies were composite in nature, including assessments conducted by the same healthcare providers responsible for prescribing dalbavancin regarding the resolution of signs and symptoms.

As shown in Tables 1 and 2, 5/9 studies recorded a clinical failure rate of less than 10%, consistent with the findings reported by Fazili et *al.* in their review. However, our analysis identified three studies, specifically by de Pablo-Mirò, Suárez and Dinh, which documented higher clinical failure rates of 50%, 22.7% and 27.8%, respectively [17, 18, 20]. It is noteworthy that one of the studies reporting a clinical failure rate below 10% did not clearly specify the follow-up duration for patients with IE [23]. It is important to highlight that the majority of the patients included in the studies were treated with a course of i.v. antibiotics before switching to dalbavancin. This suggests that the studies selected populations of patients who had already survived the initial, more critical phase of IE treatment. This selection bias could partially explain the relatively low rate of clinical failure reported in the included studies.

We found a case of resistance development, probably due to an inadequate source control. There are other cases described in literature of dalbavancin resistance development [26–29]. Notably, Cepeda et *al.* documented a case of a strain of methicillin-resistant *S. epidermidis* (MRSE) which developed resistance to dalbavancin, vancomycin, and oritavancin after a three-month course of dalbavancin, despite initially being susceptible to dalbavancin and vancomycin, with an associated teicoplanin minimum inhibitory concentration (MIC) greater than 8 µg/ml. Dalbavancin was administered at a regimen of 1500 mg every 2 weeks for the suppressive antibiotic therapy of an aortic PVE without valve replacement surgery [29]. These reports suggest additional caution in using dalbavancin as the sole agent for long-term suppressive therapy, in situations where complete source control has not been achieved.

Suarez et al. reported clinical failure in five patients, with four out of these five cases involving IE caused by *E. faecalis.* According to in vitro studies, dalbavancin shows good minimum inhibitory concentrations (MIC_50/90_) against vancomycin-susceptible *E. faecalis* [4, 30–32]. However, it is important to note that, unlike other Gram-positive cocci, the European Committee on Antimicrobial Susceptibility Testing (EUCAST) has not yet published clinical breakpoints for dalbavancin against enterococci [33]. Moreover clinical failure and the development of reduced susceptibility to dalbavancin in *E. faecalis*infections have been reported in the literature [34, 35]. Further studies are needed to evaluate the clinical effectiveness of dalbavancin for the treatment of IE caused by *E. faecalis*.

The included studies were assessed by two different tools according to their study design. We identified two studies that exhibited a serious risk of bias based on the ROBINS-I tool, whereas the six non-comparative studies included in the review were assessed as having a fair to good quality. It is important to note, however, that even if it is the recommended tool for the quality assessment of observational cohort studies without a comparator group, the Newcastle-Ottawa Scale could overestimate the quality of the studies, as it may be somewhat approximate in the assessment of certain biases, such as selection bias. In most studies incorporated into the analysis, the follow-up period commenced at the initial administration of dalbavancin, often following a course of classic intravenous antibiotic therapy for IE. This approach may introduce a notable selection bias in these studies by favouring patients who likely exhibited improved conditions, having already survived the initial days or weeks of treatment.

Despite the above discussed strengths of this review, our work has some limitations. Firstly, our review includes only observational studies. The lack of clinical trials makes it impossible to draw definitive conclusions regarding the efficacy of dalbavancin for the treatment IE. Furthermore, due to the nature of the included studies, we decided to use two different methods of quality assessment. As previously mentioned, it is essential to acknowledge that the Newcastle Ottawa Scale may potentially overestimate the quality of these studies. Another limitation of this systematic review is the unfeasibility of conducting a meta-analysis, due to the absence of clinical trials in the available literature and the high heterogeneity among the studies, which would have made the meta-analysis non-informative.

### Conclusions and future outlook

Based on the findings of this systematic review, dalbavancin might emerge as a promising and likely efficacious choice for the consolidation therapy of IE, especially in case of NVE and when the causative agent is a CoNS. Nevertheless, it should be acknowledged that the available evidence is of uncertain quality, derived from observational studies that exhibit various biases, including selection bias. Moreover, a significant portion of these studies lacks a control group.

For these reasons, dalbavancin should be used with caution when treating IE in clinical practice. Indeed, according to our findings, current literature does not provide enough data about CIED-related IE treated with dalbavancin, especially in case of IE caused by pathogens other than CoNS. There are some concerns regarding the use of dalbavancin for long-term suppressive antibiotic therapy in case of inadequate source control, which should be assessed by specific studies. In this scenario, the use of therapeutic drug monitoring could serve as a valuable aid in order to maintain the dalbavancin blood concentration above the microorganism MIC during therapy [36].

Therefore, conducting clinical trials or large observational studies specifically focused on dalbavancin’s use in IE, incorporating a control group, commencing follow-up after the first 2 weeks of intravenous therapy and clearly defining different outcomes for patients undergoing consolidation therapy versus long-term suppressive therapy, could prove invaluable for a more comprehensive assessment of dalbavancin’s efficacy in IE treatment when compared to the current SoC.

## Electronic supplementary material

Below is the link to the electronic supplementary material.

Supplementary Material 1

## Data Availability

Data is provided within the manuscript or supplementary information files.
