# Supplementary material for: A systematic review of dalbavancin efficacy as a sequential therapy for infective endocarditis
Source: Infection. 2024 Sep 26;53(1):15–23. doi: 10.1007/s15010-024-02393-9 (PMC11825564; doi:10.1007/s15010-024-02393-9)
Supplement: Supplementary file 1 — Supplementary Material 1 [file 15010_2024_2393_MOESM1_ESM.docx]

Table S1 Quality assessments for cohort studies included (NEWCASTLE-OTTAWA QUALITY ASSESSMENT SCALE).

| Author, year | Selection | | | | Comparability | Outcome | | | Total score |
| --- | --- | --- | --- | --- | --- | --- | --- | --- | --- |
|  | Representativeness  of the exposed cohort | Selection  of the non-  exposed cohort | Ascertainment  of exposure | Demonstration  that outcome of  interest was not  present at start of study | Comparability of  cohorts on the  basis of the | Assessment  of outcome | Was follow-  up long enough for | Adequacy  of follow-up of cohorts |  |
| Hidalgo-Tenorio, 2023 | 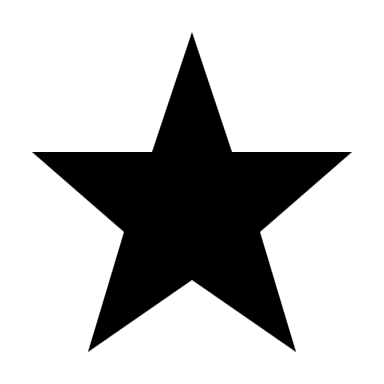 | - | 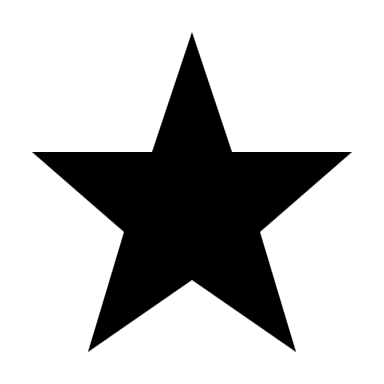 | 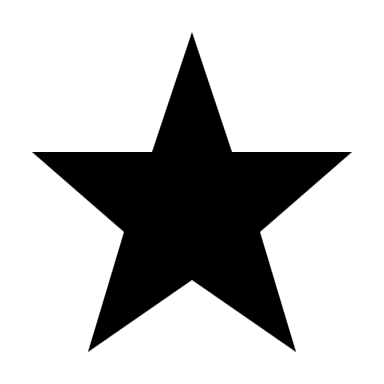 | - | 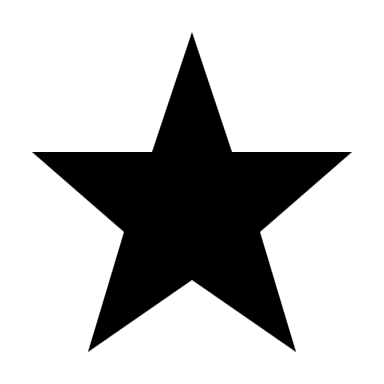 | 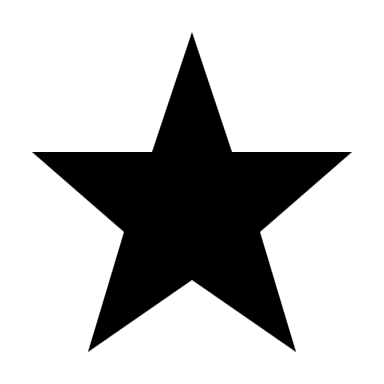 | 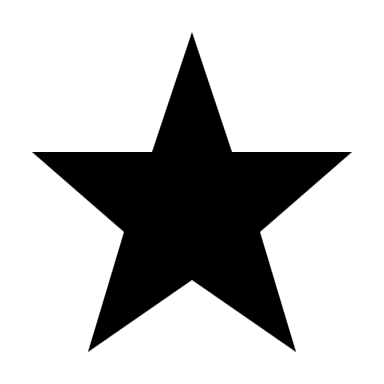 | **6/9** |
| Tobudic, 2018 | 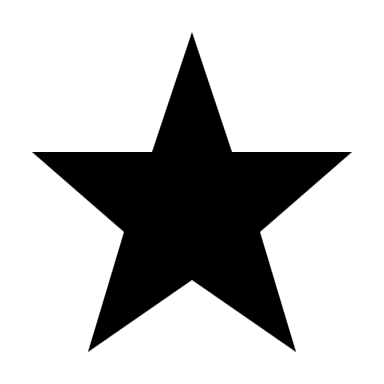 | - | 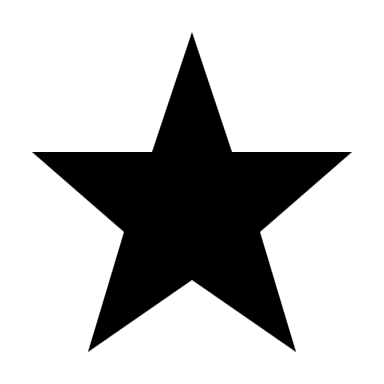 | 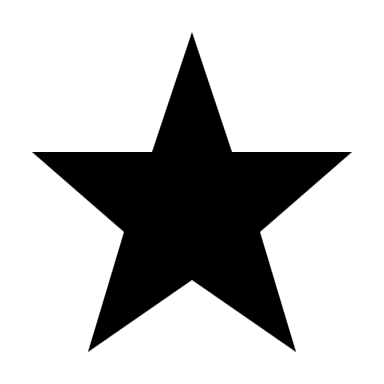 | - | 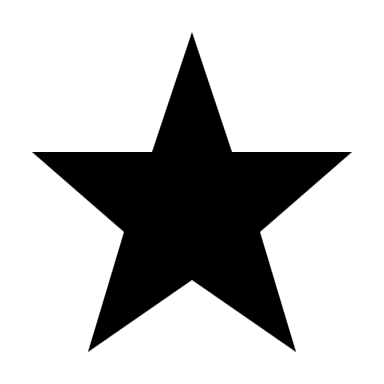 | 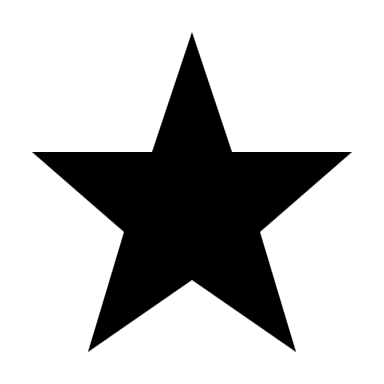 | 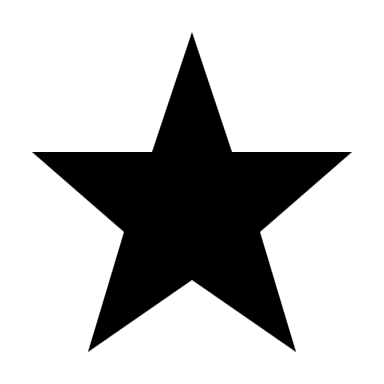 | **6/9** |
| Dihn, 2019 | 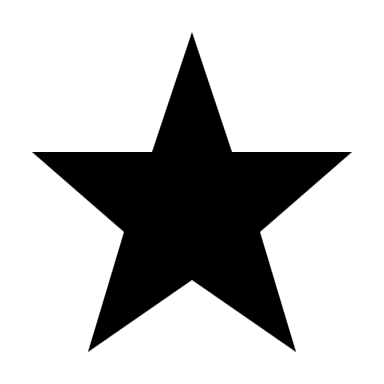 | - | 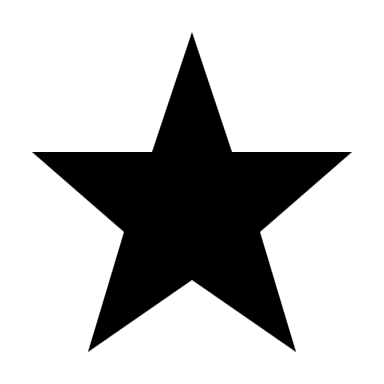 | 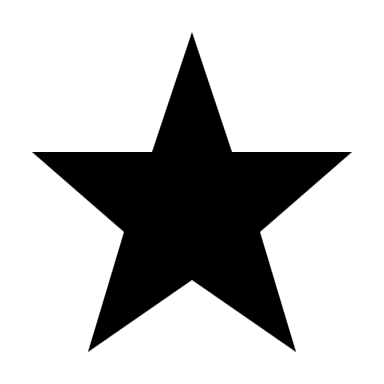 | - | 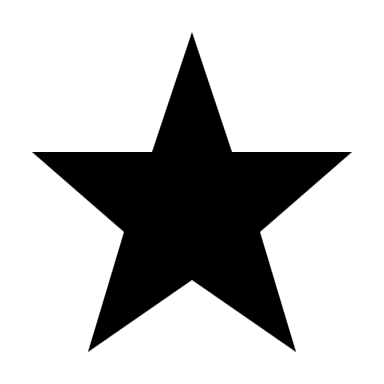 | - | 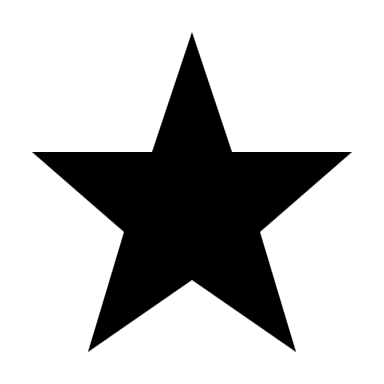 | **5/9** |
| Morata, 2022 | 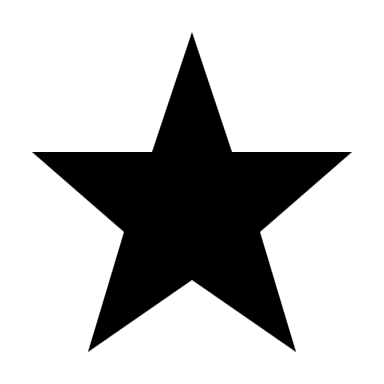 | - | 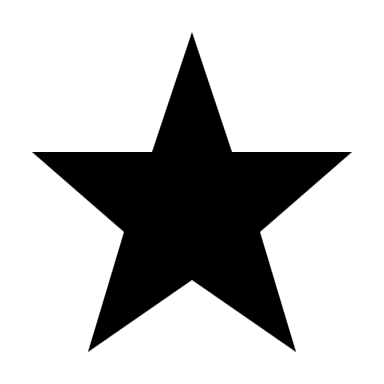 | 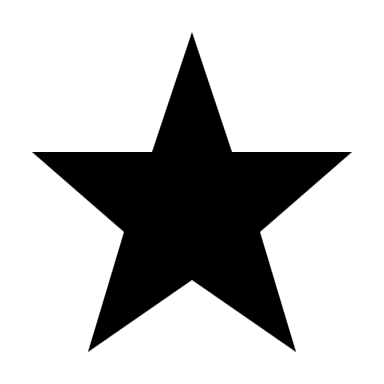 | - | 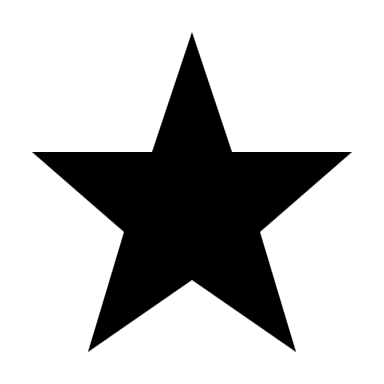 | 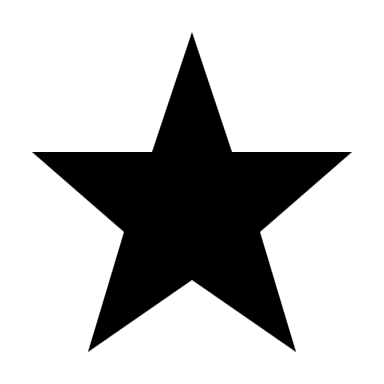 | 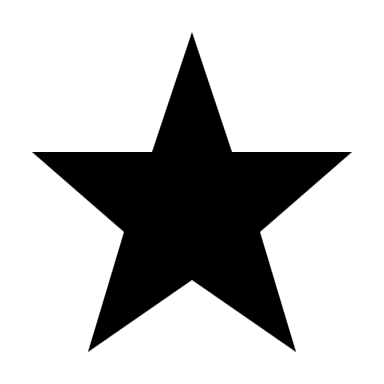 | **6/9** |
| Wunsch, 2019 | 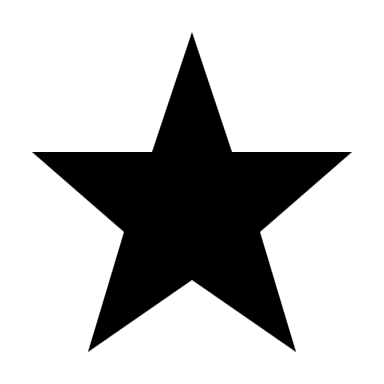 | - | 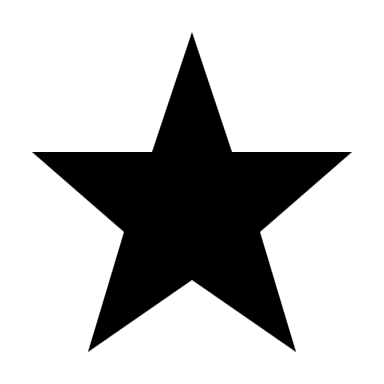 | 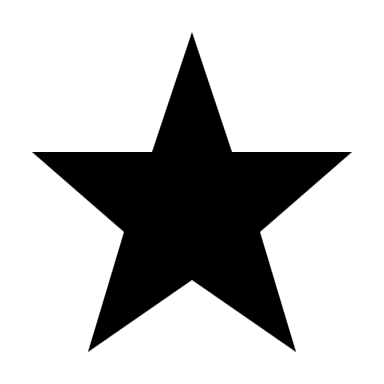 | - | 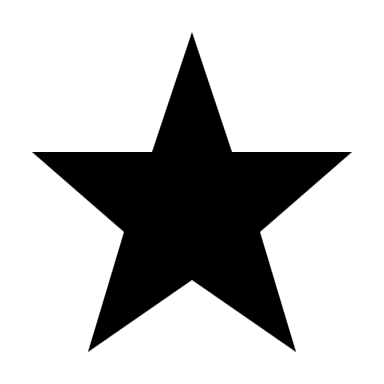 | 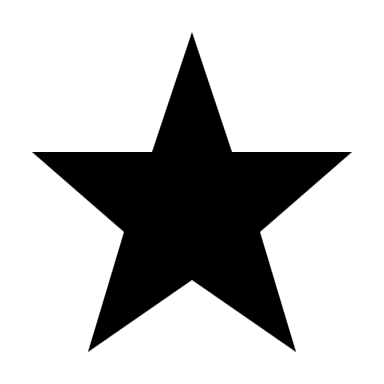 | 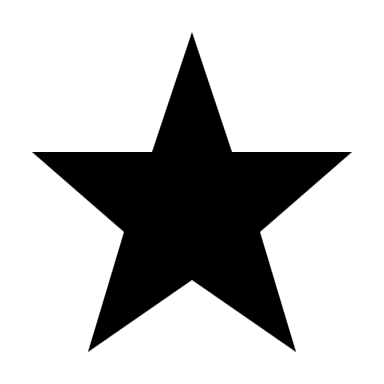 | **6/9** |
| Courjon, 2023 | 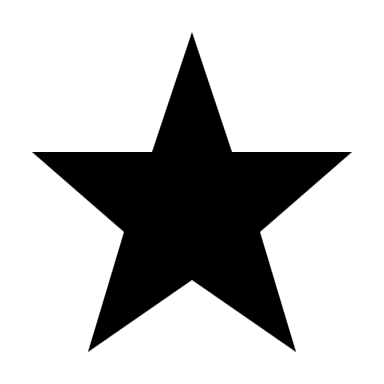 | - | 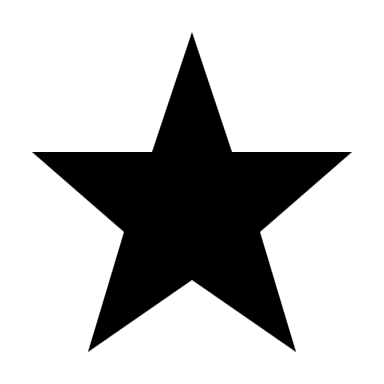 | 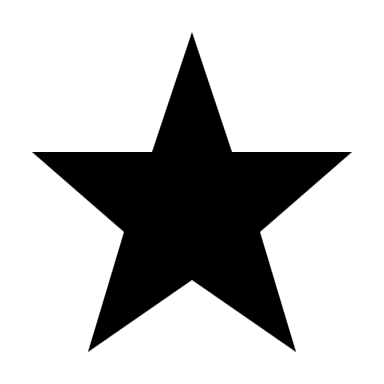 | - | 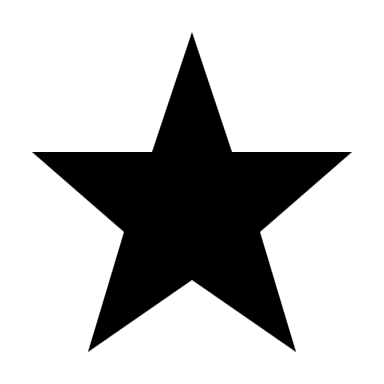 | - | 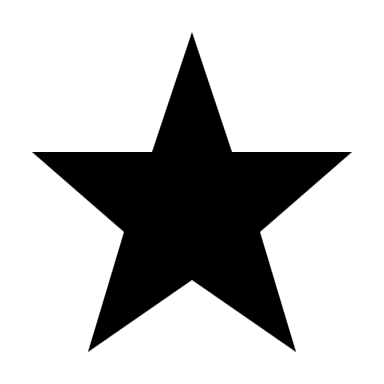 | **5/9** |

Table S2 Quality assessment (ROBINS-I tool)

| Author, year | Bias due to confounding | Bias in selection of participants into the study | Bias in classification of interventions | Bias due to deviations from intended interventions | Bias due to missing data | Bias in measurement of outcomes | Bias in selection of the reported result | Overall bias |
| --- | --- | --- | --- | --- | --- | --- | --- | --- |
| De Pablo-Miró, 2021 | moderate | moderate | low | moderate | low | moderate | low | **Moderate** |
| Veve, 2020 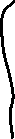 | moderate | serious | low | low | low | moderate | low | **Serious** |
| Suárez, 2023 | serious | moderate | moderate | low | low | moderate | low | **Serious** |

Table S3 Characteristics of the included studies

| Author, year | Study design | Sample size | Age (median) | Comparator group | Aim of the study | Primary outcome |
| --- | --- | --- | --- | --- | --- | --- |
| Hidalgo-Tenorio, 2023 | multicentric observational retrospective study | 124 | 67.4 | No | evaluate effectiveness of dalbavancin as consolidation therapy in IE from Gram-positive cocci | Clinical success at 12 months |
| De Pablo-Miró, 2021 | retrospective observational multicentric study (case-control) | 44 cases (8 endocarditis); 117 controls (32 endocarditis/vascular infections) | 71 | Yes | evaluate the clinical impact of the dalbavancin in comparison with other antibiotics with the same indication | Length of stay |
| Tobudic, 2018 | studio di coorte retrospettivo | 31 | 60 | No | evaluate outcomes and AE in patients with IE treated with dalbavancin | Clinical cure |
| Veve, 2020 | monocentric retrospective cohort study | 215 (57 IE) | 47 | Yes | compare the incidence of IRR between dalbavancin and SOC in Gram-positive cocci infections | Incidence of 90-d IRR (infection-related readmission) |
| Dihn, 2019 | multicentric retrospective observational study | 75 (19 IE) | 63.1 | No | describe a national French cohort of patients that have received therapy with dalbavancin | Clinical cure |
| Morata, 2022 | multicentric retrospective observational study | 197 (38 IE) | 63.9 | No | describe the sociodemographic and clinical characteristics of patients treated with dalbavancin and assess its effectiveness and safety | description of patient demographics and clinical characteristics |
| Wunsch, 2019 | multicentric retrospective observational study | 101 (25 IE) | 65 | No | evaluate the long-term indications, safety, tolerability and results of the dalbavancin | Clinical success at 90 days |
| Courjon, 2023 | prospective observational study | 151 (21 IE) | 66 | No | evaluate demographic characteristics, uses, effectiveness and safety of dalbavancin | Clinical success at 30 day |
| Suárez, 2023 | multicentric retrospective observational study | 69 (22 in the dalbavancin group vs 47 in the SoC group) | 74 (dalbavancin) vs 76 (SoC) | Yes | Analyse the experience of two Spanish hospitals in the use of dalbavancin as consolidation therapy for IE and the treatment costs compared with a group of patients with IE that received SOC | Clinical efficacy |

Table S4 Charachterics of excluded studies

| DOI | Study design | Reason for exclusion |
| --- | --- | --- |
| [10.3390/antibiotics9100700](https://doi.org/10.3390/antibiotics9100700) | retrospective observational study | Number of IE < 15 |
| 10.1080/14787210.2020.1798227 | retrospective observational study | Number of IE < 15 |
| 10.1093/ofid/ofz028 | retrospective observational study | Number of IE < 15 |
| 10.1093/ofid/ofz274 | system-wide, retrospective subanalysis | Number of IE < 15 |
| 10.1007/s40121-021-00577-6 | retrospective observational study | Wrong study design |
| 10.1017/ash.2022.229 | retrospective observational study | Number of IE < 15 |
| 10.1186/s12941-019-0329-6 | retrospective observational study | Population already included in a larger subsequent study |
| 10.1016/j.ijantimicag.2019.08.007 | retrospective observational study | Number of IE < 15 |
| [10.3390/pharmacy10010001](https://doi.org/10.3390/pharmacy10010001) | retrospective observational study | Data missing |
| [10.1093/ofid/ofac335](https://doi.org/10.1093/ofid/ofac335) | retrospective observational study | Number of IE treated with dalbavancin < 5 |

| **Section and Topic** | **Item #** | **Checklist item** | **Location where item is reported** |
| --- | --- | --- | --- |
| **TITLE** | | |  |
| Title | 1 | Identify the report as a systematic review. | Page 1, Line 1-2 |
| **ABSTRACT** | | |  |
| Abstract | 2 | See the PRISMA 2020 for Abstracts checklist. | Page 2-3, Line 25-49 |
| **INTRODUCTION** | | |  |
| Rationale | 3 | Describe the rationale for the review in the context of existing knowledge. | Page 3-4, Line 52-77 |
| Objectives | 4 | Provide an explicit statement of the objective(s) or question(s) the review addresses. | Page 3-4, Line 72-77 |
| **METHODS** | | |  |
| Eligibility criteria | 5 | Specify the inclusion and exclusion criteria for the review and how studies were grouped for the syntheses. | Page 5-6, Line 95-106 |
| Information sources | 6 | Specify all databases, registers, websites, organisations, reference lists and other sources searched or consulted to identify studies. Specify the date when each source was last searched or consulted. | Page 5, Line 87-92 |
| Search strategy | 7 | Present the full search strategies for all databases, registers and websites, including any filters and limits used. | Supplementary materials-Search strategy.pdf |
| Selection process | 8 | Specify the methods used to decide whether a study met the inclusion criteria of the review, including how many reviewers screened each record and each report retrieved, whether they worked independently, and if applicable, details of automation tools used in the process. | Page 6, Line 106-111 |
| Data collection process | 9 | Specify the methods used to collect data from reports, including how many reviewers collected data from each report, whether they worked independently, any processes for obtaining or confirming data from study investigators, and if applicable, details of automation tools used in the process. | Page 6, Line 112-116 |
| Data items | 10a | List and define all outcomes for which data were sought. Specify whether all results that were compatible with each outcome domain in each study were sought (e.g. for all measures, time points, analyses), and if not, the methods used to decide which results to collect. | Page 6, Line 114-116 |
|  | 10b | List and define all other variables for which data were sought (e.g. participant and intervention characteristics, funding sources). Describe any assumptions made about any missing or unclear information. | Page 6, Line 112-114 |
| Study risk of bias assessment | 11 | Specify the methods used to assess risk of bias in the included studies, including details of the tool(s) used, how many reviewers assessed each study and whether they worked independently, and if applicable, details of automation tools used in the process. | Page 6, Line 119-127 |
| Effect measures | 12 | Specify for each outcome the effect measure(s) (e.g. risk ratio, mean difference) used in the synthesis or presentation of results. | Page 7, Line 128-131 |
| Synthesis methods | 13a | Describe the processes used to decide which studies were eligible for each synthesis (e.g. tabulating the study intervention characteristics and comparing against the planned groups for each synthesis (item #5)). | Page 7, Line 129-131 |
|  | 13b | Describe any methods required to prepare the data for presentation or synthesis, such as handling of missing summary statistics, or data conversions. | Not applicable |
|  | 13c | Describe any methods used to tabulate or visually display results of individual studies and syntheses. | Page 7, Line 130-131 |
|  | 13d | Describe any methods used to synthesize results and provide a rationale for the choice(s). If meta-analysis was performed, describe the model(s), method(s) to identify the presence and extent of statistical heterogeneity, and software package(s) used. | Not applicable |
|  | 13e | Describe any methods used to explore possible causes of heterogeneity among study results (e.g. subgroup analysis, meta-regression). | Not applicable |
|  | 13f | Describe any sensitivity analyses conducted to assess robustness of the synthesized results. | Not applicable |
| Reporting bias assessment | 14 | Describe any methods used to assess risk of bias due to missing results in a synthesis (arising from reporting biases). | Page 6, Line 119-127 |
| Certainty assessment | 15 | Describe any methods used to assess certainty (or confidence) in the body of evidence for an outcome. | Not applicable |
| **RESULTS** | | |  |
| Study selection | 16a | Describe the results of the search and selection process, from the number of records identified in the search to the number of studies included in the review, ideally using a flow diagram. | Page 7, Line 135-140, Figure 1 |
|  | 16b | Cite studies that might appear to meet the inclusion criteria, but which were excluded, and explain why they were excluded. | Table S4 |
| Study characteristics | 17 | Cite each included study and present its characteristics. | Table 1, Table S3 |
| Risk of bias in studies | 18 | Present assessments of risk of bias for each included study. | Table S1 and S3 |
| Results of individual studies | 19 | For all outcomes, present, for each study: (a) summary statistics for each group (where appropriate) and (b) an effect estimate and its precision (e.g. confidence/credible interval), ideally using structured tables or plots. | Not applicable |
| Results of syntheses | 20a | For each synthesis, briefly summarise the characteristics and risk of bias among contributing studies. | Bias assessment results |
|  | 20b | Present results of all statistical syntheses conducted. If meta-analysis was done, present for each the summary estimate and its precision (e.g. confidence/credible interval) and measures of statistical heterogeneity. If comparing groups, describe the direction of the effect. | Page 7-8, Line 149-167 |
|  | 20c | Present results of all investigations of possible causes of heterogeneity among study results. | Not applicable |
|  | 20d | Present results of all sensitivity analyses conducted to assess the robustness of the synthesized results. | Not applicable |
| Reporting biases | 21 | Present assessments of risk of bias due to missing results (arising from reporting biases) for each synthesis assessed. | Table S1-S2 |
| Certainty of evidence | 22 | Present assessments of certainty (or confidence) in the body of evidence for each outcome assessed. | Not applicable |
| **DISCUSSION** | | |  |
| Discussion | 23a | Provide a general interpretation of the results in the context of other evidence. | Page 12-14, Line 253-315 |
|  | 23b | Discuss any limitations of the evidence included in the review. | Page 14, Line 316-318 |
|  | 23c | Discuss any limitations of the review processes used. | Page 14, Line 316-324 |
|  | 23d | Discuss implications of the results for practice, policy, and future research. | Page 14-15, Line 326-344 |
| **OTHER INFORMATION** | | |  |
| Registration and protocol | 24a | Provide registration information for the review, including register name and registration number, or state that the review was not registered. | Page 4, Line 79-80 |
|  | 24b | Indicate where the review protocol can be accessed, or state that a protocol was not prepared. | Page 4, Line 80 |
|  | 24c | Describe and explain any amendments to information provided at registration or in the protocol. | Page 6, Line 122-127 |
| Support | 25 | Describe sources of financial or non-financial support for the review, and the role of the funders or sponsors in the review. | Page 15, Line 347 |
| Competing interests | 26 | Declare any competing interests of review authors. | Page 15, Line 353 |
| Availability of data, code and other materials | 27 | Report which of the following are publicly available and where they can be found: template data collection forms; data extracted from included studies; data used for all analyses; analytic code; any other materials used in the review. | Supplementary materials (“data extraction”) |
